# Comparison of extraction sites versus artificial defects with xenogenic bone substitute in minipigs

**DOI:** 10.1002/cre2.390

**Published:** 2021-01-04

**Authors:** Constanze Steiner, Matthias Karl, Matthias W. Laschke, Peter Schupbach, Andrea Venturato, Angelines Gasser

**Affiliations:** ^1^ Department of Prosthodontics Saarland University Homburg Germany; ^2^ Institute for Clinical and Experimental Surgery Saarland University Homburg Germany; ^3^ Laboratory for Histology, Electron Microscopy and Imaging Schupbach Ltd. Thalwil Switzerland; ^4^ Nobel Biocare Holding AG Kloten Switzerland

**Keywords:** artificial site, bone substitute material, extraction site, socket preservation

## Abstract

**Objectives:**

The preclinical evaluation of bone substitutes is frequently performed in artificially created defects. However, such defects do not reflect the predominant clinical application of bone substitutes for socket preservation. Hence, the goal of this animal study was to compare the performance of a xenogenic bone substitute in extraction sites versus artificial defects.

**Material and Methods:**

Four study sites each were created in the mandibles of four minipigs in the region of the third premolars and first molars, respectively. On one side, fresh extraction sockets were established while contralaterally trephine defects were created in healed alveolar bone. All sites were augmented using a particulate xenogenic bone substitute, covered by resorbable membranes and allowed to heal for 12 weeks. The amounts of new bone, non‐bone tissue and remaining bone substitute granules were quantified through histological and micro‐CT analysis. Comparative statistics were based on *t*‐tests for two samples and ANOVA with the level of significance set at *α* = 0.05.

**Results:**

Histomorphometric data from only two animals could be quantitatively analyzed due to difficulty with identifying the surgical sites. The percentage of newly formed bone ranged between 53.2% ± 5.6% for artificial defects and 54.9% ± 12.4% for extraction sites. With the exception of ANOVA indicating a greater amount of non‐bone tissue in extraction sites as compared to artificial sites (*p* = 0.047), no statistically significant differences were observed. Micro‐CT scans showed patterns similar to the ones observed in histomorphometry. As extraction sites could be identified only in two micro‐CT reconstructions, quantitative assessment was not undertaken.

**Conclusions:**

Despite the comparable performance of bone substitute material in artificial defects and extraction sites found here, the data gathered with this experiment was insufficient for showing equivalence of both approaches.

## 1 INTRODUCTION

Over the past years, several strategies aimed at shortening overall treatment times in implant dentistry have been developed. While the concept of immediate implant placement is still under debate (Chen et al., 2004; Chrcanovic et al., 2015; Khouly & Keenan, 2015), there seems to be consensus that bone resorption following tooth extraction should be prevented (Beck & Mealey, 2010; Mardas et al., 2010) in order to avoid major reconstructions of alveolar bone prior to implant placement (Probst et al., 2020).

Tooth extraction has been shown to trigger a cascade of events ultimately leading to healing of hard and soft tissues (Amler, 1969; Amler et al., 1960; Boyne, 1966). This cascade comprises three distinct phases of inflammation, proliferation and modeling/remodeling (Araújo, Silva, et al., 2015). From a clinical perspective, socket healing causes substantial alterations in alveolar ridge morphology leading to a reduction in vertical and horizontal dimensions (Araújo, Silva, et al., 2015; Misawa et al., 2016). Such changes may not only make implant placement impossible (Block et al., 2002) but may also lead to limitations with respect to function and esthetics (Artzi & Nemcovsky, 1998; Barone et al., 2008; Kotsakis et al., 2016; Schropp et al., 2003).

A variety of techniques has been described for preventing alveolar bone loss including the application of different biomaterials as well as combinations thereof (De Coster et al., 2011). Guided bone regeneration (GBR) procedures (Buser et al., 1998) involving particulate biomaterials in combination with a membrane for maintaining space and for hindering faster growing soft tissue from invading the bone space have been advocated (Turri et al., 2016). In addition, Turri and coworkers demonstrated a bioactive effect for specific membrane types (Turri et al., 2016). While the results presented for GBR procedures are not uniform, most authors conclude that less resorption takes place when socket preservation procedures are being performed (Araújo, da Silva, et al., 2015; Barone et al., 2008; Jung et al., 2013; Lekovic et al., 1997; Oltramari et al., 2007). While bone quantity may be better preserved, the mechanical quality of the regenerated bone has been reported as being compromised (De Coster et al., 2011; Horváth et al., 2013) with fibrous tissue surrounding bone substitute materials especially in the coronal part of a socket (Mardas et al., 2010). It has also been reported that the addition of bone substitute material into extraction sockets may even delay healing (Araújo & Lindhe, 2009; Jensen et al., 2006).

Recent animal research provided evidence that stem cells present in the periodontal ligament (PDL) not only govern the biologic response of the PDL to mechanical stimuli (Huang et al., 2016) but may also positively affect bone healing even when only parts of the extraction socket remain covered with PDL remnants (Pei et al., 2017; Yuan et al., 2018). On the contrary, the use of drills for creating osteotomies for dental implant placement have been shown to create a zone of dying cells surrounding an osteotomy (Chen et al., 2018) which may negatively impact osseous regeneration.

So far, biomaterials for bone augmentation have been predominantly tested in artificially created defects of a critical size (Ma et al., 2009; Schlegel et al., 2006) which, even in the case of using large size animal models, may have been located extraorally (Buser et al., 1998; Jensen et al., 2006; Schlegel et al., 2006). Only few authors reported on socket grafting in an animal model (Indovina Jr. & Block, 2002; Kunert‐Keil et al., 2015). Given that particulate bone substitute materials are predominantly used in extraction sockets, results obtained in non‐extraction sites seem doubtful as the animal models utilized for evaluating the potential of a specific biomaterial should mimic their clinical use (Li et al., 2015).

Based on these considerations, the goal of this animal study was to establish an extraction socket‐based minipig model to assess the performance of a well‐described xenogenic bone substitute in a GBR procedure and directly compare it to the performance in artificially created defects (Pawlowsky et al., 2017).

## 2 MATERIAL AND METHODS

### 2.1 Ethical statement

This study was approved by the local governmental animal protection committee (Landesamt für Verbraucherschutz des Saarlandes; permission number: 19/2018) and conducted in accordance with the Directive 2010/63/EU and the NIH Guidelines for the Care and Use of Laboratory Animals (NIH Publication #85‐23 Rev. 1985). A total of four adult (minimum age 24 months) Aachen minipigs (Pawlowsky et al., 2017) were used for this study.

### 2.2 Surgical interventions

All animals underwent two surgical interventions in the mandible, which were carried out as follows. After 12 h of fasting, the animals were sedated using an intramuscular injection of ketamine (Ketavet; 30 mg/kg bodyweight; Zoetis, Parsippany, NJ), xylazine (Rompun; 2.5 mg/kg bodyweight; Bayer Vital GmbH, Leverkusen, Germany) and atropin (Atropinsulfat; 1 mg; B. Braun Melsungen AG, Melsungen, Germany) followed by the application of a permanent venous catheter in the animals' ears for fluid substitution (0.9% NaCl). After endotracheal intubation, general anesthesia was maintained using 2% isoflurane (Portec, GME 3; Fritz Stephan GmbH, Gackenbach, Germany), while continuously monitoring vital parameters (Guardian, RS Meditec Healthcare GmbH, Duisburg, Germany). The perioral skin was shaved and disinfected using iodine while the body of the animal was covered by sterile drapes. Both at the beginning and at the end of each surgical intervention, subcutaneous injections of buprenorphine (Temgesic; 0.025 mg/kg bodyweight; Indivior UK Ltd., Slough/Berkshire, UK) were administered and two fentanyl patches (release rate 100 μg/h over a 72 h period) were attached on the animals' backs (Fentanyl Hennig; Hennig Arzneimittel, Flörsheim am Main, Germany). Additionally, all animals received single shot antibiotics (Naxcel; 100 mg/mL; Zoetis, Parsippany, NJ) through intramuscular injections.

On postoperative days 3 and 10, general anesthesia was again induced as described above for inspecting and cleaning the surgical sites (Chlorhexamed; GlaxoSmithKline Consumer Healthcare, Munich, Germany) or for removing the sutures. Postoperatively, the animals were kept on a soft diet until suture removal. For termination of the experiment, all animals were again anesthetized and sacrificed by intracardial injection of T61 (0.12 ml/kg body weight; Merck Animal Health, Madison, NJ). Mandibular block sections containing the surgical sites were obtained removing all soft tissue and fixed in neutrally buffered formalin.

The first intervention included the unilateral extraction of the third and fourth premolars as well as the first molar in a randomized manner (simple randomization by coin flipping). Following local anesthesia (Ultracain D‐S forte; 1:100 000, Sanofi‐Aventis, Frankfurt, Germany), the teeth were cleaned using a piezosurgery unit (Piezomed, W&H, Bürmoos, Austria). After intrasulcular incisions had been made, full‐thickness mucoperiosteal flaps were reflected, the multi‐rooted teeth were sectioned using a high‐speed contra angle and carbide burrs. Extractions were then completed using elevators, forceps and piezosurgery where needed. Prior to primary wound closure using simple interrupted and horizontal mattress sutures (Supramid 4–0; Resorba Medical GmbH, Nuremberg, Germany), alveolar bone height was reduced, and periosteal releasing incisions were made for achieving tension‐free closure. Intraoral radiographs (Heliodent, Dentsply Sirona, York, PA) were taken in order to verify complete removal of all roots.

**FIGURE 1:**
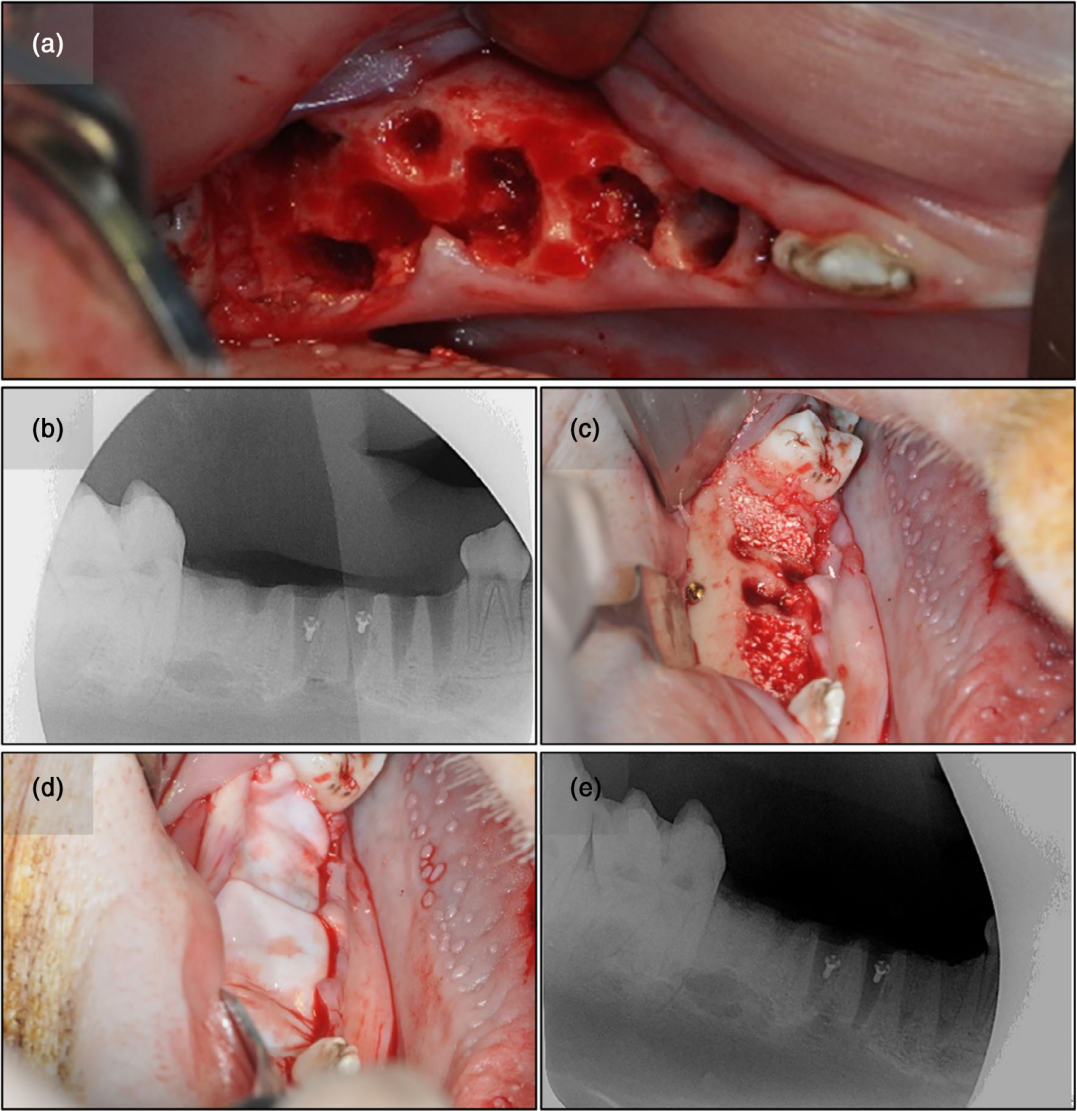
Clinical situation of an extraction site after removal of the third and fourth premolars and the first molar (a). Complete tooth removal was verified radiographically (b) before the defects were augmented with particulate biomaterial (c) and covered with a resorbable membrane (d). The postoperative radiograph (e) shows the alveolae filled with bone substitute material and osteosynthesis screws placed for orientation purposes

**FIGURE 2:**
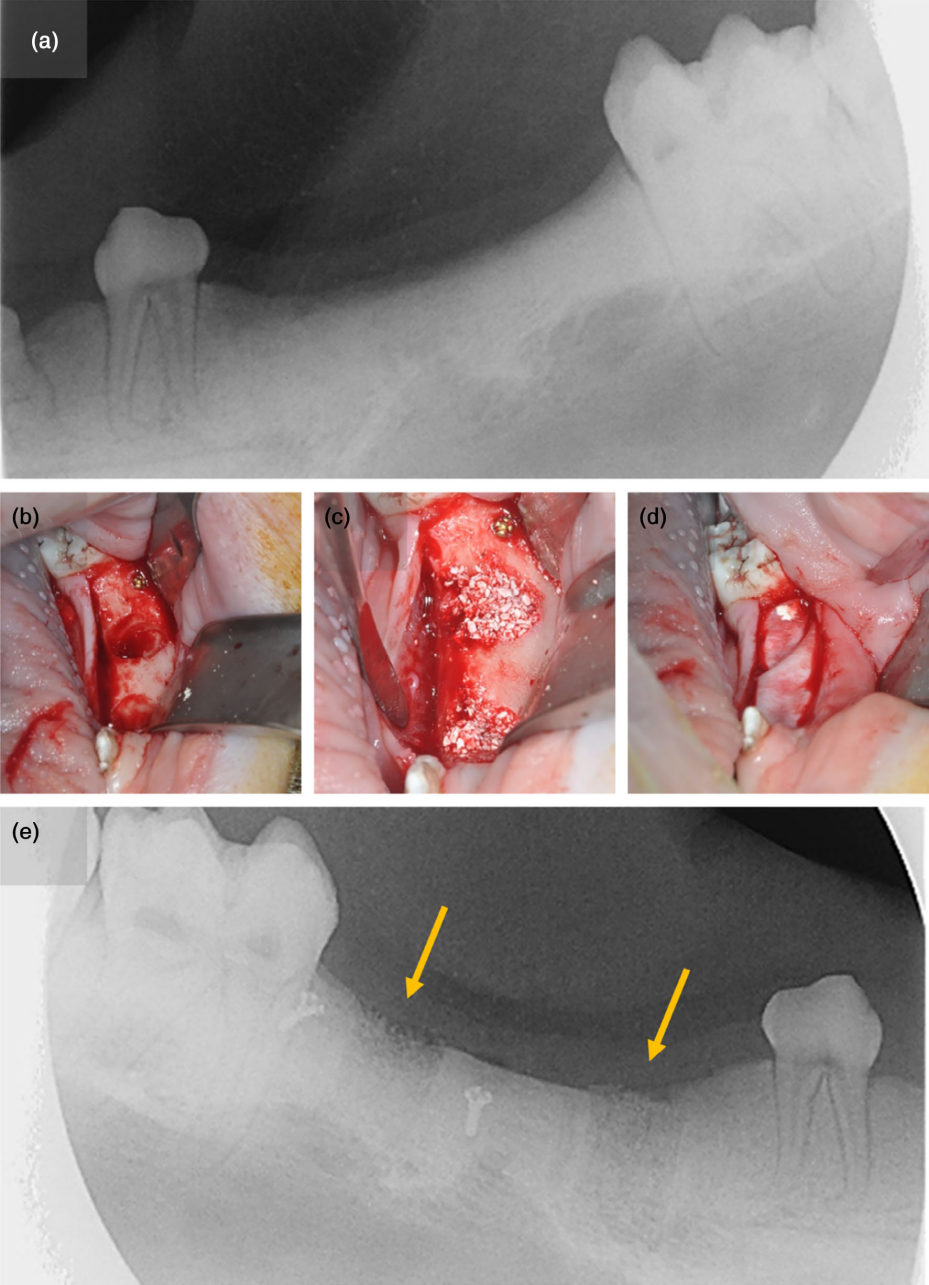
Radiograph of an artificial site after 12 weeks of healing following extraction of the third and fourth premolars and the first molar (a). Two intrabony defects were created using trephine burrs (b) before the defects were augmented with particulate biomaterial (c) and covered with a resorbable membrane (d). The postoperative radiograph (e) shows the defects filled with bone substitute material (yellow arrows) and osteosynthesis screws placed for orientation purposes

Following 12 weeks of healing (Buser et al., 1998; Kunert‐Keil et al., 2015), two standardized intrabony defects were created in the region of the third premolar and the first molar using trephine burrs with a diameter of 7 mm, while the depths of the defects were determined so as not to damage the alveolar canal (artificial sites). The defects were filled with particulate xenogenic bone substitute material (creos xenogain, Nobel Biocare AB, Gothenburg, Sweden) and covered by a xenogenic, resorbable collagen membrane (creos xenoprotect, Nobel Biocare AB) without using membrane fixation pins. Osteosynthesis screws (Stryker, Duisburg, Germany) were placed for identifying the surgical sites after healing (Lekovic et al., 1997). On the contralateral side of the mandible, the third and fourth premolars as well as the first molars were extracted, and the sockets filled using the biomaterials described above (extraction sites). Following periosteal releasing incisions, simple interrupted and horizontal mattress sutures (Supramid 4–0; Resorba Medical GmbH) were used for achieving tension‐free closure both in the artificial defects and in the extraction sites. Intraoral radiographs were obtained to verify a complete fill of the defects as well as to determine the positions of the surgical sites relative to the osteosynthesis screws. The augmented sites were again allowed to heal for 12 weeks. Representative pictures of the two procedures, are shown in Figures 1 and 2.

### 2.3 Histologic preparation

The specimens were placed in 10% buffered formalin for at least 3–5 days and sent to a commercial research laboratory for histology, electron microscopy and imaging (Schupbach Ltd., Thalwil, Switzerland) to be prepared for light microscopy according to the cutting‐grinding technique (Donath & Breuner, 1982) and for micro‐CT scanning. The specimens were washed in 0.01 M phosphate buffered saline (PBS buffer; Sigma‐Aldrich Chemie GmbH, Buchs, Switzerland) and dehydrated for 4 days in each step in an ascending series of an ethanol‐pure water series (60%, 80% and 96%) with the final step being in absolute ethanol (Sigma‐Aldrich Chemie GmbH). As soon as the bone specimen had reached the 70% ethanol bath, they were evaluated by multi‐slice micro‐CT analysis (micro‐CT 40; Scanco Medical AG, Brüttisellen, Switzerland). Afterwards, the specimens were infiltrated with a graded series of ethanol and Technovit 7200 VLC (Kulzer, Wehrheim, Germany) embedding resin over a period of at least 12 days at standard temperature and constant shaking. Subsequently, the specimens were placed in three consecutive containers of 100% Technovit 7200 VLC for 24 h. Following dehydration and infiltration the specimens were placed into embedding molds filled with fresh Technovit 7200 VLC and polymerized by 450 nm light for 10 h, using a light polymerization unit (Exakt Apparatebau, Norderstedt, Germany), while cooling with running tap water to avoid temperatures exceeding 40°C.

The polymerized specimens were then sliced in the buccal‐lingual direction using a diamond band saw (Exakt Apparatebau, Norderstedt, Germany). These sections of approximately 200 μm thickness were reduced by microgrinding and polishing (Exakt Apparatebau) to an even thickness of 60–80 μm. Final polish was applied with 0.1 μm diamond polishing paste. The sections were stained using Sanderson's RBS stain (Dorn and Hart, Villa Park) and counter‐stained using acid fuchsin.

Sections were cover slipped for analysis and for each hemi‐mandible four to six histological sections were collected and imaged (Leica M205A/Leica DM6B, LEICA Microsystems GmbH, Wetzlar, Germany).

### 2.4 Histomorphometry

Histomorphometric analysis was performed using both a Leica M205A stereo light microscope and a Leica DM6B light microscope (LEICA Microsystems GmbH, Wetzlar, Germany), a microscope digital camera system (Leica 490) and a PC‐based image analysis system (IMS, Imagic, Glattbrugg, Switzerland). The analysis included the determination of the parameters percentage of new bone formation, percentage of remaining graft granules and percentage of non‐bone components (connective tissue and empty space).

Each histological section was imaged, and where possible the region of interest (ROI; i.e., the area contained by the socket walls or artificial defect walls) was identified and measured (mm^2^), next the respective areas of graft granules and non‐bone components were measured, and the area of the new bone was calculated as the remaining area when compared to the total ROI using IMS software (Imagic, Glattbrugg, Switzerland). For each animal, the two histological sections of extraction site and artificial site with the largest ROI were selected on the assumption that a larger analysis area is more representative of the granules, bone and non‐bone distribution. Histological quantification of the percentage of area of granules, bone and non‐bone component was carried out on 18 (out of 39) sections while quantification was not possible for the remaining 21 sections due to challenges in the identification of the ROI and of the bone substitute granules.

A t‐test for two samples with unequal variance was performed for each one of the three groups (granules, bone and non‐bone tissue) with the level of significance set at *α* = 0.05 (Microsoft Excel, Analysis ToolPak add‐in). The test was selected due to the low amount of available datapoints and the unknown variance for the Artificial sites and Extraction sites. Data analysis was performed by selecting for each animal's mandible and type of surgical site the two histological sections with the greatest ROI and statistical comparison was carried out as described above. Furthermore, in addition to *t*‐test, a one‐way ANOVA test was performed for each one of the three groups to support statistical evaluation.

### 2.5 Micro‐CT analysis

Micro‐CT scanning was performed on all samples using a high‐resolution micro‐CT 40 (Scanco Medical AG, Brüttisellen, Switzerland) with an *x*‐, *y*‐, *z*‐resolution of 20 μm. Each image data set consisted of approximately 400–600 micro‐CT X‐ray images. The original grayscale images were processed with a slight Gaussian low pass filtration for noise reduction and a fixed segmentation threshold to separate bone from graft particles. The image data sets were used to calculate the volume (%) of bone and graft particles and to produce 3‐D views of the specimens using specific software (Scanco Medical AG).

## 3 RESULTS

The experimental part of this study was completed uneventfully with the following exceptions. In one animal (animal 2), a major cyst was discovered in the molar region during extraction as part of the first surgical intervention. While the cyst could be enucleated, postoperative inflammation was observed. During the second intervention, a similar situation was encountered on the contralateral side, which led to the decision to only augment the alveolae of the third premolar. Following bone augmentation, inflammatory reactions were encountered in two animals with one artificial site and one extraction site being affected. These complications were resolved successfully by local interventions, that is, debridement and rinsing with chlorhexidine.

**FIGURE 3:**
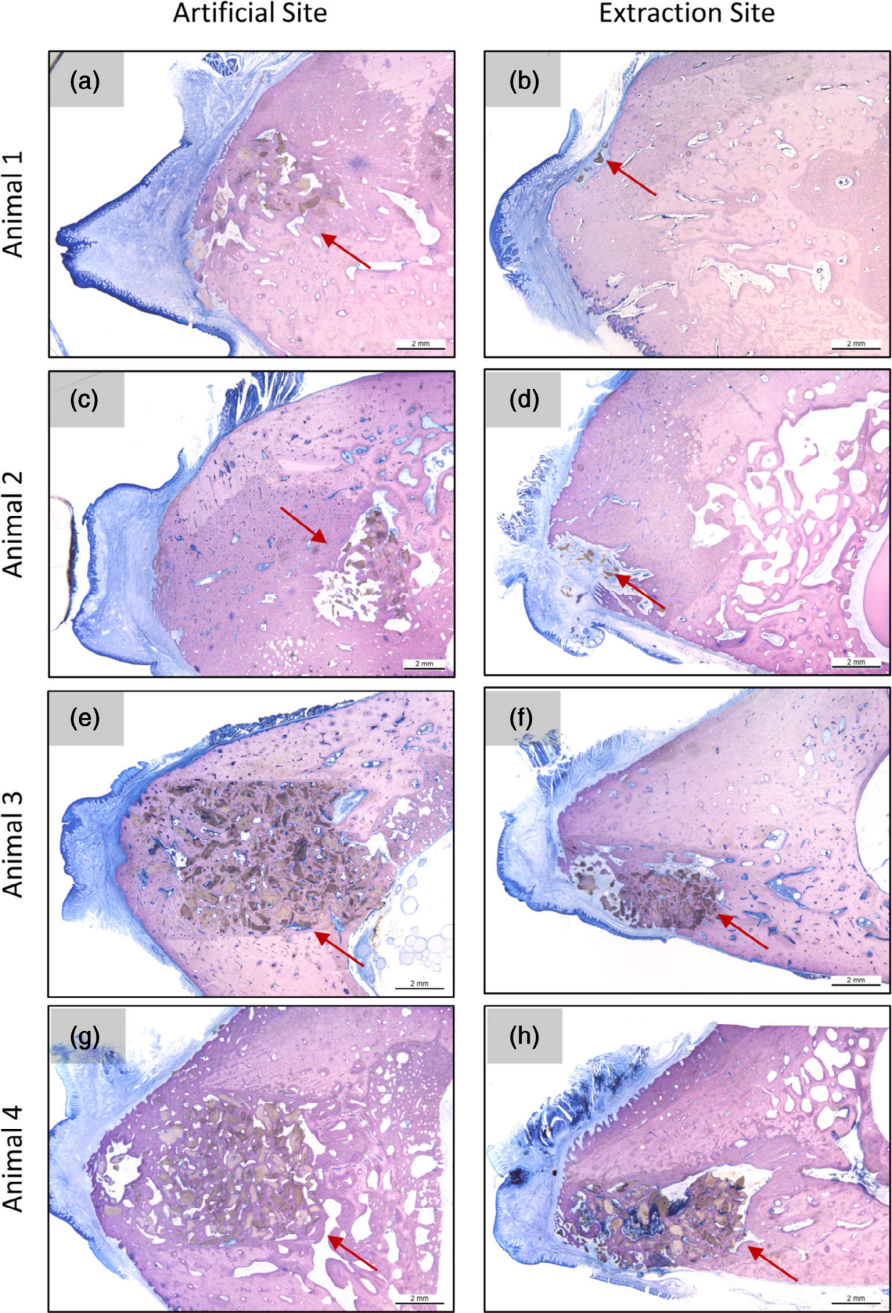
(a) and (b) histological section of animal 1 showing representative images of artificial site (1‐L‐section 4) and extraction site (1‐R‐section 4), respectively. (c) and (d) histological section of animal 2 showing representative images of artificial site (2‐L‐section 4) and extraction site (2‐R‐section 2), respectively. (e) and (f) histological section of animal 3 showing representative images of artificial site (3‐R‐section 6) and extraction site (3‐L‐section 1), respectively. (g) and (h) histological section of animal 4 showing representative images of artificial site (4‐R‐section 5) and extraction site (4‐L‐section 3), respectively. Red arrows indicate the presence of bone substitute granules. Scale bars = 2 mm. Images courtesy of Schupbach Ltd

**FIGURE 4:**
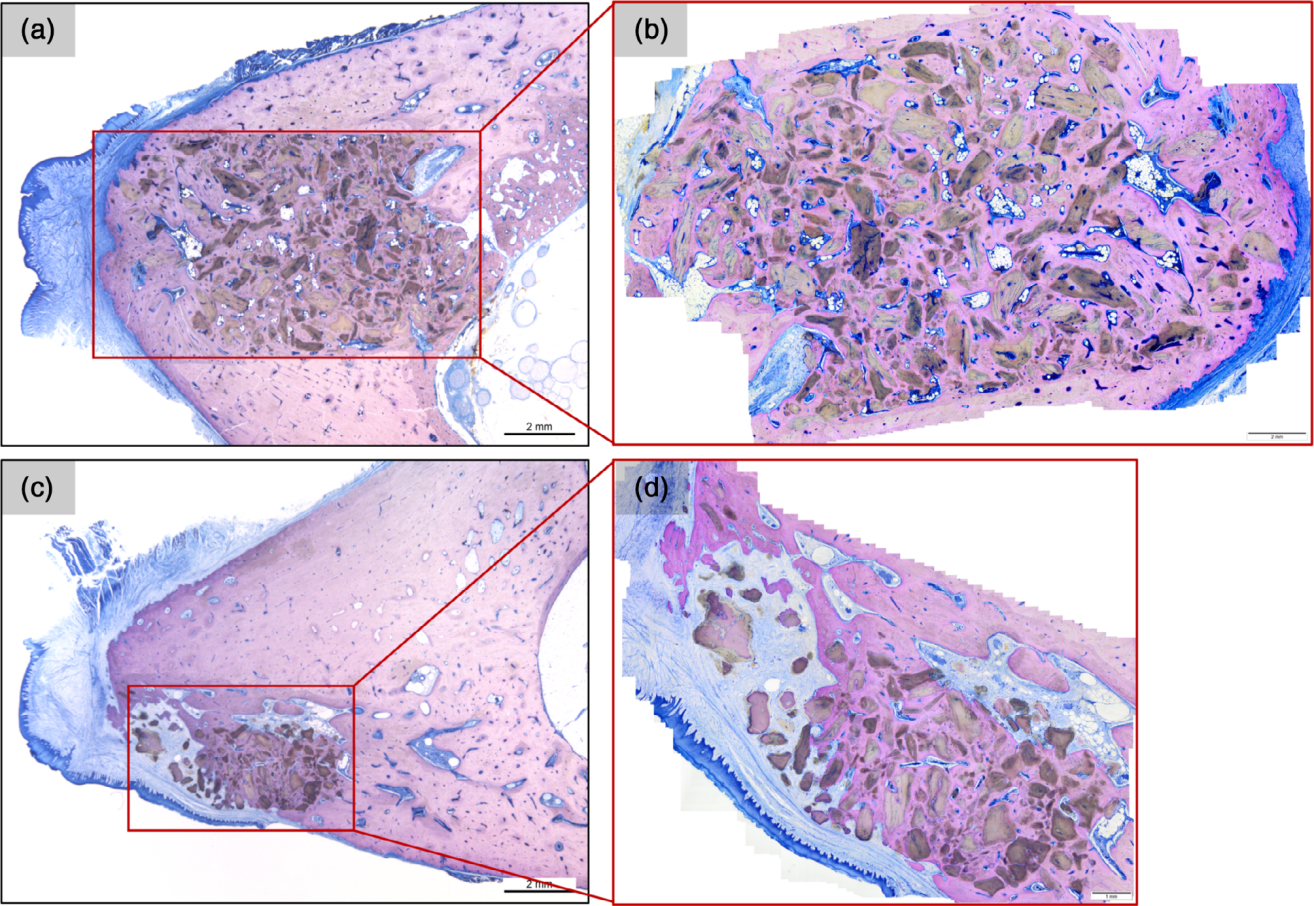
(a) Representative histological section of artificial site (3‐R‐section 6) in animal 3 and (b) higher magnification image of area of analysis for the artificial site (3‐R‐section 6). Note: Image (b) is rotated clockwise 180°. (c) Representative histological section of extraction site (3‐L‐section 1) in animal 3 and (d) close up and area of analysis for the extraction site (3‐L‐section 1). Scale bars = 2 mm. Images courtesy of Schupbach Ltd

Representative histological sections of the four extraction sites and of the four artificial sites are given in Figure 3. Overall, the histological sections of animals 3 and 4 showed a higher number of granules, while the percentages of bone and non‐bone tissue were similar to those observed in animal 2. Large variations in the percentages of bone and non‐bone tissue were observed in animal 1. Furthermore, in animals 3 and 4, considerable differences in granule distribution were observed between artificial sites and extraction sites (Figure 4).

For animal 1, it was not possible to perform histomorphometric analysis on the extraction site since none of the histological sections revealed the presence of bone substitute granules (exception: very few granules in section 1‐R‐section 4). Similarly, for animal 2 it was not possible to perform histomorphometric analysis on the extraction site due to the reduced amount of bone substitute granules identified which were mostly embedded in connective tissue. Consequently, data analysis was only performed on a total of four sections for the extraction site and four sections for the artificial site obtained from animals 3 and 4 (Table 1).

**TABLE 1:** Summary of the fraction (%) of area occupied by granules, bone and non‐bone tissue used for statistical analysis

	Granules area [%]		Bone area [%]		Non‐bone tissue area [%]	
	Extraction	Artificial	Extraction	Artificial	Extraction	Artificial
Animal 3	25.74	35.74	48.23	45.54	26.03	18.72
	5.50	33.24	69.89	53.93	24.65	12.83
Animal 4	19.19	21.96	43.09	58.58	37.72	19.46
	17.42	27.2	62.52	55.52	20.06	17.28

Comparing the three parameters (granules, bone, non‐bone tissue) using *t*‐test with unequal variance (Table S1), no differences were observed in the percentage of bone, which was 53.16% ± 5.58% for artificial site and 54.89% ± 12.42% for extraction site. Small but non‐significant differences were observed in the percentage of granules between extraction sites and artificial sites, with the artificial sites showing higher percentage of granules (29.02% ± 6.19%) compared to the extraction sites (14.75% ± 8.44%). Similarly, non‐significant differences were observed in the percentage of non‐bone tissue, with 16.86% ± 2.97% for the artificial sites and 26.40% ± 7.52% for the extraction sites. ANOVA (Table S2) also showed no statistical differences for granules and bone but a weak statistical difference for non‐bone tissue (*p* = 0.047). However, when evaluating the finding on non‐bone tissue, the low number of datapoints available and the consequent low statistical power should be taken into account. Quantification of bone density on the buccal and lingual aspects of the ROI was not possible due to the quality of the samples.

**FIGURE 5:**
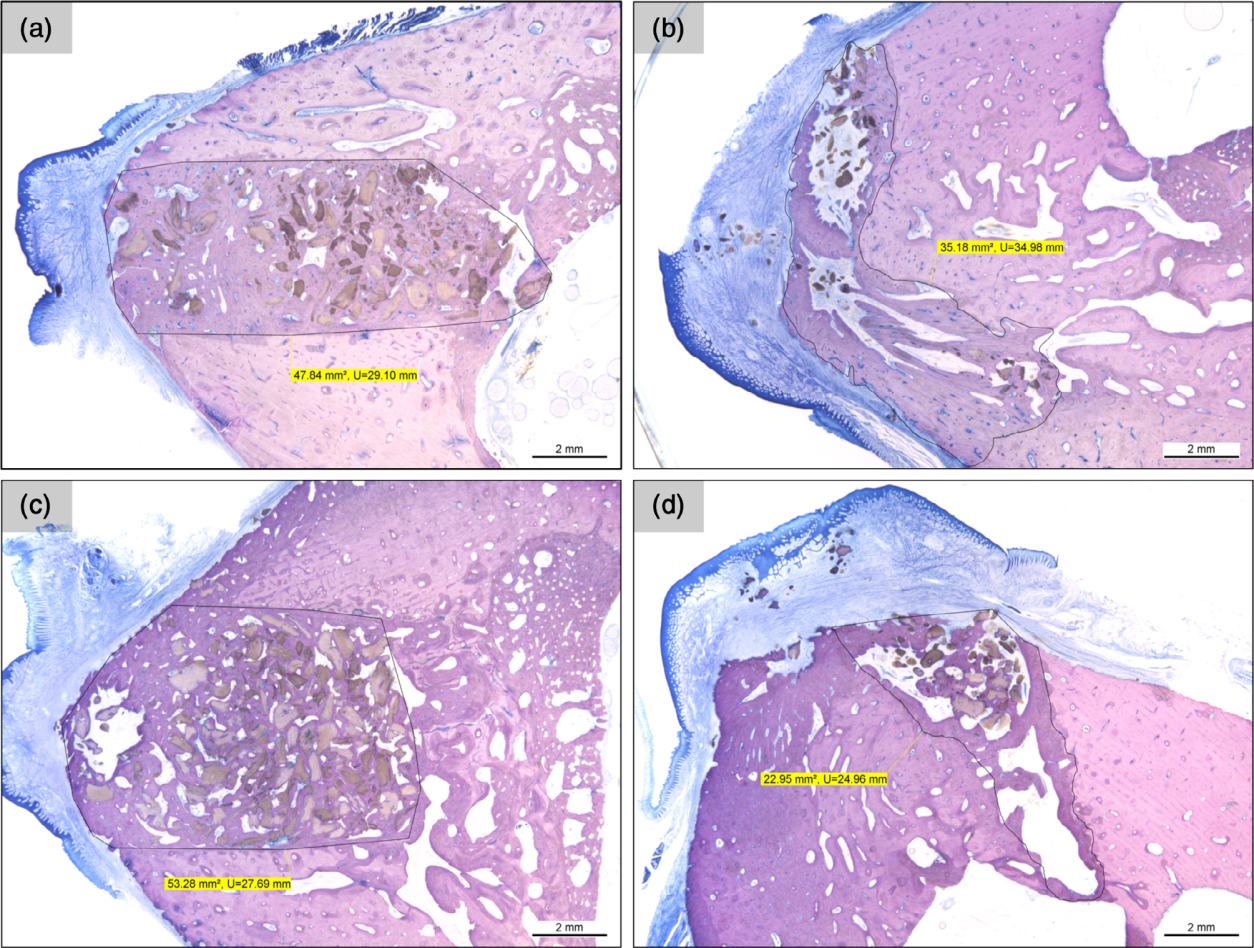
(a) Histological section of the artificial site (3‐R‐section 5) in animal 3 and its ROI identification and quantification. (b) Histological section of the extraction site (3‐L‐section 5) in animal 3 and its ROI identification and quantification. (c) Histological section of the artificial site (4‐R‐section 5) in animal 3 and its ROI identification and quantification. (d) Histological section of the extraction site (4‐L‐section 6) in animal 3 and its ROI identification and quantification. Scale bars = 2 mm. Images courtesy of Schupbach Ltd

Despite the mostly non‐significant differences between artificial and extraction sites, it was possible to notice that the standard deviation for the calculated values was consistently smaller for the artificial site as compared to the extraction site suggesting that the artificial site allows for more homogeneous results. Additionally, a qualitative observation of the ROIs for extraction and artificial sites showed that the artificial sites preparation allows for an easier and more accurate ROI identification as shown in Figure 5.

**FIGURE 6:**
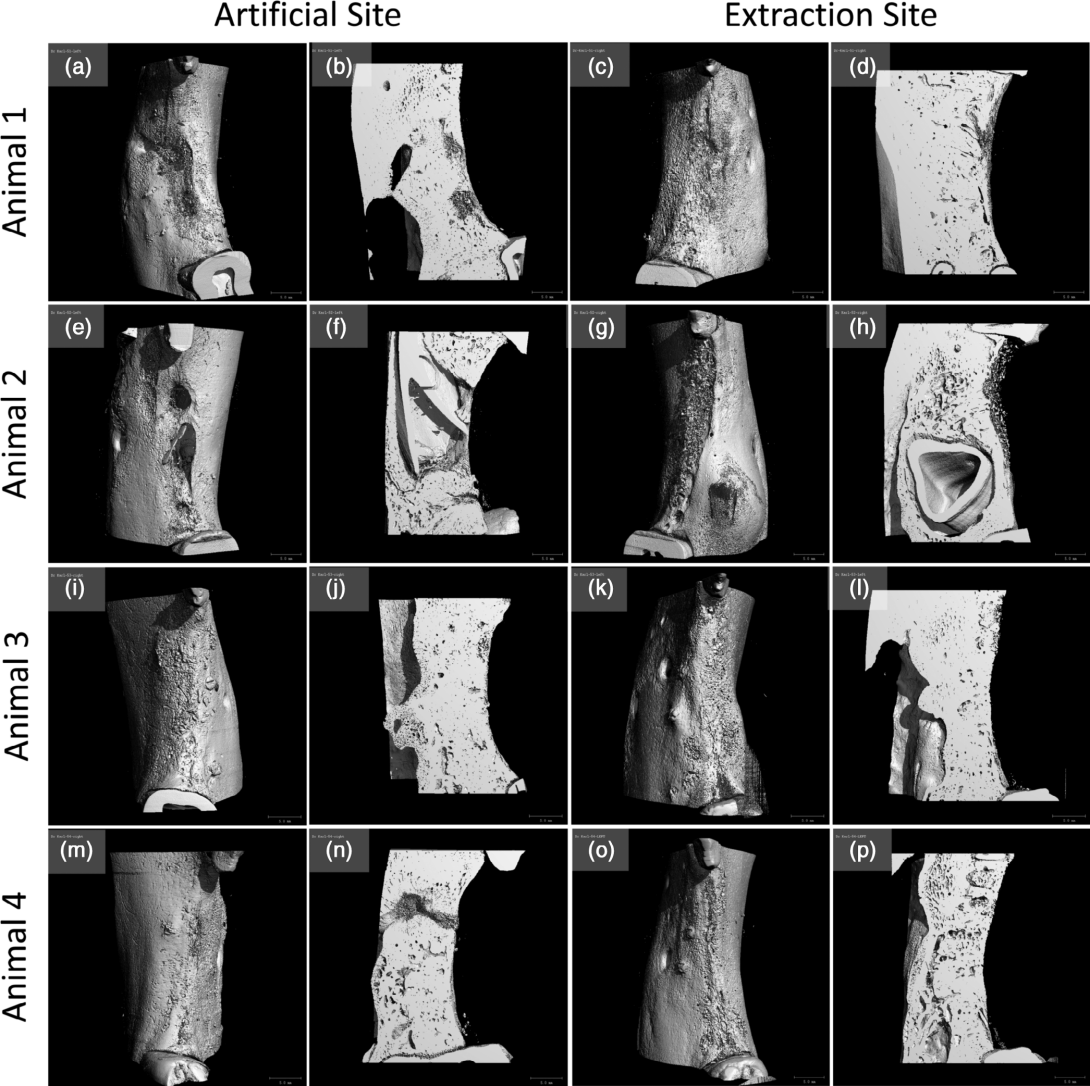
(a) and (b) represent the 3D reconstruction and a cross‐section of the artificial site for animal 1, respectively. (c) and (d) represent the 3D reconstruction and a cross‐section of the extraction site for animal 1, respectively. (e) and (f) represent the 3D reconstruction and a cross‐section of the artificial site for animal 2, respectively. (g) and (h) represent the 3D reconstruction and a cross‐section of the extraction site for animal 2, respectively. (i) and (j) represent the 3D reconstruction and a cross‐section of the artificial site for animal 3, respectively. (k) and (l) represent the 3D reconstruction and a cross‐section of the extraction site for animal 3, respectively. (m) and (n) represent the 3D reconstruction and a cross‐section of the artificial site for animal 4, respectively. (o) and (p) represent the 3D reconstruction and a cross‐section of the extraction site for animal 4, respectively. Images courtesy of Schupbach Ltd

Micro‐CT scanning allowed for building 3D reconstructions (Figure 6) out of which only seven could be correctly assessed while for others it was not possible to discriminate between local bone and newly formed bone. The available reconstructions showed situations comparable to those observed in the histomorphometric analysis with respect to the three parameters granules, bone and non‐bone tissue. The higher degree of variability observed may be due to the small number of samples and to the difficulty to precisely outline the ROIs.

## 4 DISCUSSION

While not reflecting a clinically relevant condition, creating standardized bony defects (Ma et al., 2009; Schlegel et al., 2006) as done here by using trephine burrs allows for quantitative histomorphometric comparisons e.g. among different bone substitute materials. Extractions sites (Indovina Jr. & Block, 2002; Kunert‐Keil et al., 2015) on the other hand cannot be standardized in a porcine animal model characterized by fragile multi‐rooted teeth, which are prone to fracture during extraction. As a consequence, the extraction sites were characterized by areas covered with remnants of the periodontal ligament and areas which had been altered using hand instruments, burrs and piezosurgery, respectively (Pei et al., 2017; Yuan et al., 2018). Additionally, the amount of bone substitute material needed for augmentation as well as the level of condensation of the biomaterial could not be standardized.

With the primary goal of comparing the performance of a specific bone substitute material in different types of defects, a control group where no biomaterial was added (Mardas et al., 2003) has not been considered. From a methodological point of view, the addition of a group with empty defects ensuring that the defect size realized constituted a critical size defect (Probst et al., 2020; Ruehe et al., 2009; Shanbhag et al., 2017) would have been desirable.

With primary wound closure constituting a prerequisite for bone augmentation, substantial vertical reduction of alveolar bone as well as periosteal releasing incisions were required. This drastically limited the vertical height of both the extraction sockets and the artificially created defects and has to be seen as a further limitation of this animal model. Despite these measures, the problems encountered in finding the defects and the bone substitute granules in the histologic sections may have been due to materials exposure following suture removal with the granules being displaced.

Quantitative analysis did not show differences in the amount of newly formed bone and bone substitute granules remaining in artificial sites and extraction sites after healing. For non‐bone tissue, the t‐test with unequal variance showed no statistically significant difference, while ANOVA showed a weak but significant difference with greater amounts of non‐bone tissue being present in extraction sites. ANOVA results should be further validated in future studies given the low statistical power of the current assessment. With this finding, it can only be speculates that remnants of the periodontal ligament had only a limited or even no effect on bone regeneration at 12 weeks. The histological findings seen here seem to be in line with previous reports on human biopsies following alveolar ridge preservation procedures where new bone formation in the apical parts and fibrous connective tissue in the coronal parts of the sockets have been described (Mardas et al., 2010).

Despite the lack of quantifiable data, qualitative observation of the histological sections suggests that the artificial site preparation could be a useful approach for the development of a standardized study design to assess the performance of bone substitutes. Artificial sites were easier to identify, and the region of interests were better defined and easier to outline during analysis.

The low number of specimens analyzed in this study precludes drawing final conclusions on the impact of the two site preparation methods on materials' performance. Further investigations with a much greater sample size would be required for answering this question. In the light of the technical limitations encountered, both clinically and analytically, it appears that well‐defined artificial defect models are more reliable.

## CONFLICT OF INTEREST

The authors C.S., P.S. and M.W.L. declare that they have no conflict of interest. The author M.K. reports grants from Nobel Biocare Services AG, during the conduct of the study. The author A.V. is currently working at Nobel Biocare AG as R&D Manager Regeneratives & Biologics. The author A.G. is an employee of Nobel Biocare Services, which financed the study.

## AUTHOR CONTRIBUTION

Conceptualization, C.S., M.K. and A.G.; methodology, M. W. L. and P. S.; formal analysis, M. W. L. and P. S.; data curation, C.S., P. S. and A. G.; writing—original draft preparation, M.K., A.G., A.V.; writing—review and editing, A.V. All authors have read and agreed to the published version of the manuscript.

## Supporting information

**Table S1** Summary of the results of t‐test for two samples assuming unequal variance (α = 0.05). For each group (granules, bone and non‐bone tissue) a test was carried out to compare extraction and artificial sites. N = 2 per animal. Total N = 4. No statistical differences were identified between Artificial and Extraction site for the three groups.
**Table S2:** Summary of the results of the one‐way ANOVA test (α = 0.05). For each group (granules, bone and non‐bone tissue) a test was carried out to compare Artificial and Extraction sites. N = 2 per animal. Total N = 4. No statistical differences were identified between Artificial and Extraction sites for the granules and bone groups. Non‐bone tissue group showed weak statistical differences (p = 0.047), however the low amount of datapoints do not allow for sufficient statistical power to confirm the finding. SS = sum‐of‐squares, df = degree of freedom, MS = mean square, F = F ratio.

## Data Availability

Research data are not shared.
