# Supplementary material for: Comparison of extraction sites versus artificial defects with xenogenic bone substitute in minipigs
Source: Clin Exp Dent Res. 2021 Jan 4;7(4):490–501. doi: 10.1002/cre2.390 (PMC8404495; doi:10.1002/cre2.390)
Supplement: Supplementary file 1 — Table S1 Summary of the results of t‐test for two samples assuming unequal variance (α = 0.05). For each group (granules, bone and non‐bone tissue) a test was carried out to compare extraction and artificial sites. N = 2 per animal. Total N = 4. No statistical differences were identified between Artificial and Extraction site for the three groups. Table S2: Summary of the results of the one‐way ANOVA test (α = 0.05). For each group (granules, bone and non‐bone tissue) a test was carried out to compare Artificial and Extraction sites. N = 2 per animal. Total N = 4. No statistical differences were identified between Artificial and Extraction sites for the granules and bone groups. Non‐bone tissue group showed weak statistical differences (p = 0.047), however the low amount of datapoints do not allow for sufficient statistical power to confirm the finding. SS = sum‐of‐squares, df = degree of freedom, MS = mean square, F = F ratio. [file CRE2-7-490-s001.docx]

**Appendix**

**Table S1:** Summary of the results of t-test for two samples assuming unequal variance (α=0.05). For each group (granules, bone and non-bone tissue) a test was carried out to compare extraction and artificial sites. N = 2 per animal. Total N = 4. No statistical differences were identified between Artificial and Extraction site for the three groups.

| **t-Test: Two-Sample Assuming Unequal Variances** | | | | | | | | |
| --- | --- | --- | --- | --- | --- | --- | --- | --- |
| **Granules** | | | **Bone** | | | **Non-bone tissue** | | |
|  | *Extraction* | *Artificial* |  | *Extraction* | *Artificial* |  | *Extraction* | *Artificial* |
| Mean | 16.9625 | 29.535 | Mean | 55.9325 | 53.3925 | Mean | 27.115 | 17.0725 |
| Variance | 71.20149 | 38.35397 | Variance | 154.1551 | 31.12903 | Variance | 56.4975 | 8.818758 |
| Observations | 4 | 4 | Observations | 4 | 4 | Observations | 4 | 4 |
| Hypothesized Mean Difference | 0 |  | Hypothesized Mean Difference | 0 |  | Hypothesized Mean Difference | 0 |  |
| df | 6 |  | df | 4 |  | df | 4 |  |
| t Stat | -2.40234 |  | t Stat | 0.373203 |  | t Stat | 2.485199 |  |
| P(T<=t) two-tail | 0.053125 |  | P(T<=t) two-tail | 0.727934 |  | P(T<=t) two-tail | 0.067832 |  |
| t Critical two-tail | 2.446912 |  | t Critical two-tail | 2.776445 |  | t Critical two-tail | 2.776445 |  |
| NON-SIGNIFICANT DIFFERENCES | | | NON-SIGNIFICANT DIFFERENCES | | | NON-SIGNIFICANT DIFFERENCES | | |

**Table S2:** Summary of the results of the one-way ANOVA test (α=0.05). For each group (granules, bone and non-bone tissue) a test was carried out to compare Artificial and Extraction sites. N = 2 per animal. Total N = 4. No statistical differences were identified between Artificial and Extraction sites for the granules and bone groups. Non-bone tissue group showed weak statistical differences (p=0.047), however the low amount of datapoints do not allow for sufficient statistical power to confirm the finding. SS = sum-of-squares, df = degree of freedom, MS = mean square, F = F ratio.

| **One Way Anova for Granules** | | | | | | |
| --- | --- | --- | --- | --- | --- | --- |
| SUMMARY |  |  |  |  |  |  |
| *Site* | *Count* | *Sum* | *Average* | *Variance* |  |  |
| Extraction | 4 | 67.85 | 16.9625 | 71.20149167 |  |  |
| Artificial | 4 | 118.14 | 29.535 | 38.35396667 |  |  |
| ANOVA |  |  |  |  |  |  |
| *Source of Variation* | *SS* | *df* | *MS* | *F* | *P-value* | *F crit* |
| Between Sites | 316.1355125 | 1 | 316.1355125 | 5.771241658 | 0.053125178 | 5.987377607 |
| Within Sites | 328.666375 | 6 | 54.77772917 |  |  |  |
| Total | 644.8018875 | 7 |  |  |  |  |

| **One Way Anova for Bone** | | | | | | |
| --- | --- | --- | --- | --- | --- | --- |
| SUMMARY |  |  |  |  |  |  |
| *Site* | *Count* | *Sum* | *Average* | *Variance* |  |  |
| Extraction | 4 | 223.73 | 55.9325 | 154.1550917 |  |  |
| Artificial | 4 | 213.57 | 53.3925 | 31.129025 |  |  |
| ANOVA |  |  |  |  |  |  |
| *Source of Variation* | *SS* | *df* | *MS* | *F* | *P-value* | *F crit* |
| Between Sites | 12.9032 | 1 | 12.9032 | 0.139280152 | 0.721827692 | 5.987377607 |
| Within Sites | 555.85235 | 6 | 92.64205833 |  |  |  |
| Total | 568.75555 | 7 |  |  |  |  |
| **One Way Anova for Non-bone Tissue** | | | | | | |
| SUMMARY |  |  |  |  |  |  |
| *Site* | *Count* | *Sum* | *Average* | *Variance* |  |  |
| Extraction | 4 | 108.46 | 27.115 | 56.4975 |  |  |
| Artificial | 4 | 68.29 | 17.0725 | 8.818758333 |  |  |
| ANOVA |  |  |  |  |  |  |
| *Source of Variation* | *SS* | *df* | *MS* | *F* | *P-value* | *F crit* |
| Between Sites | 201.7036125 | 1 | 201.7036125 | 6.176214549 | 0.047469833 | 5.987377607 |
| Within Sites | 195.948775 | 6 | 32.65812917 |  |  |  |
| Total | 397.6523875 | 7 |  |  |  |  |
